# How Do Trainees Use EPAs to Regulate Their Learning in the Clinical Environment? A Grounded Theory Study

**DOI:** 10.5334/pme.1403

**Published:** 2024-09-05

**Authors:** Bart P. A. Thoonen, Nynke D. Scherpbier-de Haan, Cornelia R. M. G. Fluit, Renée E. Stalmeijer

**Affiliations:** 1 Development of Education in Primary Care at the Department of Primary and Community Care, Radboud University Medical Centre, PO Box 9101, 6500 HB Nijmegen, The Netherlands; 2 Department of Primary and Long-term Care, University Medical Centre Groningen, Groningen, The Netherlands; 3 Radboud University Medical Centre, Nijmegen, The Netherlands; 4 School of Health Professions Education, Faculty of Health, Medicine and Life Sciences, Maastricht University, Maastricht, The Netherlands

## Abstract

**Introduction:**

Entrustable Professional Activities (EPAs) can potentially support self-regulated learning in the clinical environment. However, critics of EPAs express doubts as they see potential harms, like checkbox behaviour. This study explores how GP-trainees use EPAs in the clinical environment through the lens of self-regulated learning theory and addresses the question of whether EPAs help or hinder trainees’ learning in a clinical environment.

**Methods:**

Using constructivist grounded theory methodology, a purposive and theoretical sample of GP-trainees across different years of training were interviewed. Two PICTOR interviews were added to refine and confirm constructed theory. Data collection and analysis followed principles of constant comparative analysis.

**Results and Discussion:**

Trainees experience both hindering and helping influences of EPAs and self-regulate their learning by balancing these influences throughout GP-placements. Three consecutive stages were constructed each with different use of EPAs: adaptation, taking control, and checking the boxes. EPAs were most helpful in the ‘taking control’ stage. EPAs hindered self-regulated learning most during the final stage of training as trainees had other learning goals and experienced assessment of EPAs as bureaucratic and demotivating. Regularly discussing EPAs with supervisors helped to focus on specific learning goals, create opportunities for learning, and generate task-oriented feedback.

**Conclusion:**

EPAs can both help and hinder self-regulated learning. How trainees balance both influences changes over time. Therefore, placements need to be at least long enough to enable trainees to gain and maintain control of learning. Supervisors and teachers should assist trainees in balancing the hindering and helping influences of EPAs.

## Introduction

Entrustable Professional Activities (EPAs) have been introduced in many health professions education (HPE) curricula. Combined with competency-based curricula, EPAs aim to inform training and learning activities and provide a framework for assessment, feedback, and decision-making [1,2,3,4]. In workplace learning, competencies can be challenging to observe and may not offer sufficient guidance for feedback and feedforward when not directly related to the actual work [5]. By defining observable and entrustable activities, the concept of EPAs was developed to bridge the gap between competencies and real-world clinical practice [2,5]. EPAs are expected to yield benefits such as more targeted feedback and specific insights into trainees’ growth and development. However, researchers have expressed concerns about whether EPAs truly facilitate self-directed learning, as they may encourage checkbox behaviour and hinder self-regulation [6,7]. If EPAs do not meet expectations, their use and potential could be seriously limited. Therefore, evaluating trainees’ actual use of EPAs for self-regulated learning in clinical environments may help to inform future development and implementation of EPAs.

### Learning in the clinical learning environment

HPE situated in clinical environments has the potential to provide ample opportunities for learning [8,9,10]. However, the workplace is not primarily designed as a learning environment and clinical work easily interferes with the learning process [11,12]. In many specialties, turnover rates of patient encounters are high, providing numerous learning opportunities. Consequently, it can be challenging to identify and choose the most relevant learning goals to focus on [13,14]. Time constraints and constantly changing affordances may hinder learning when there is not enough time to attain one learning goal before proceeding to the next [8,13,14,15]. These circumstances make it challenging for trainees to identify what to learn, which activities to prioritize, and how to develop required competencies [12,16]. The theory of self-regulated learning can be useful as a lens to understand how trainees handle these challenges [14,16,17].

Self-regulated learning is grounded in social cognitivism and relates to the (self-) regulatory interactions between individuals and their environment to achieve personal goals [18]. Theoretical frameworks of self-regulated learning commonly used in medical education are those by Zimmerman [19] and Pintrich [20]. These frameworks share a common concept of self-regulation as a cyclical process where learners actively set goals, plan learning activities, monitor progress and evaluate goal attainment during the learning process [21]. Feedback, obtained from observations and assessments is paramount in self-regulated learning [22,23,24,25]. Promoting self-regulated learning enhances and improves academic and clinical performance and contributes to development of life-long learning professionals [9]. External regulation by assessments and assignments can positively impact self-regulated learning, particularly in complex and unstructured environments such as clinical workplaces [14,26].

EPAs, as observable tasks, can help to promote self-regulated learning as EPAs can inform learning goals and provide a task-based reference for feedback [4,6]. However, research so far has focussed predominantly on EPA development, EPA-assessment, and entrustment decisions rather than on EPAs’ impact on learning [3,15,27].

To inform future development and implementation of EPAs in the clinical learning environment, the aim of this study was to further understand and explore the mechanisms and actions postgraduate trainees apply to regulate their learning when engaging with EPAs. The main research question guiding this study was: through the lens of self-regulated learning theory, how do EPAs help or hinder trainees’ learning in a clinical environment?

## Methods

### Worldview

This study was designed from a constructivist perspective, enabling a comprehensive exploration of trainee actions, experiences, and viewpoints. The constructivist approach necessitates an exploratory and open-minded interpretation of results, aiding in theory generation to address the research question [28].

### Methodology

This study, informed by constructivist grounded theory, adopted an inductive approach with concepts from self-regulated learning [29,30]. The constructivist perspective facilitated active variation search and comparative data analysis, enabling the construction of theory on how EPAs impact trainee’s self-regulated learning.

### Context

The research was conducted in the three-year postgraduate GP-specialty training at Radboud University Medical Centre Nijmegen (RadboudUMC). The first and final years involved GP-office placements, while the second year included placements in secondary and tertiary care settings. The study focused on the GP-office placements in the first and final years.

Trainees learn through supervised work at the GP-office, supplemented by weekly academic days for experience sharing and small group learning. Trainees are expected to self-regulate their learning through feedback collection, reflection, and planning via learning goals and personal plans.

In 2017, a national set of 81 professional activities was introduced to stimulate workplace learning. From this, the Nijmegen GP-training curriculum developed and implemented 25 EPAs. These were developed by a curriculum committee, that selected activities from the national set, based on the need for intentional or externally regulated learning. The ten Cate template was used to design the final EPAs [2].

In the Nijmegen program, EPAs serve as elective learning tasks based on individual competence development and learning needs. They guide learning activity planning, observations, and feedback. Mastery of an EPA is determined by the supervisor when the appropriate level of entrustment is reached. Entrustment decisions are informed by both informal undocumented observations and workplace-based assessments (e.g. mini-CEX). As such, entrustment decisions were included as a formative endpoint to mark completion of the EPA as a learning task.

To inform competency development and high stakes progress decisions, trainees gather observations and assessments in an e-portfolio, according to the concept of programmatic assessment [31,32].

### Data collection

Using a constructivist grounded theory design, we collected data from in-depth semi-structured interviews. These interviews were chosen as they are ideal for exploring personal experiences and thoughts, and for obtaining detailed information through follow-up questions [33]. The interviews focused on how trainees self-regulate their learning and the impact of EPAs on this process. Sensitizing concepts were introduced to highlight elements of self-regulated learning such as goal setting, planning, monitoring, feedback, and reflection. An interview guide is provided in appendix 1. In constructivist grounded theory a close interaction between researcher and participants contributes to the collection of salient data and theory development [29]. Therefore, all interviews were conducted by BT as the main researcher. Memos were written to capture thoughts, ideas, and to reflect on the interaction between the interviewer and the trainee. To finalize our theory, we applied the Pictor technique in two additional interviews. The Pictor technique, as described by King et al [34] has its origins in Personal Construct Theory [Kelly 1955 as cited in 34]. This technique can be used to explore people’s construct systems and – often unconscious- thoughts and ideas about how people or things are related in daily life. We applied this technique to two trainees that recently finished their training. Based on their final training year, trainees were given cards, each displaying a factor (Table 2), and asked to arrange them on a flip-chart labelled ‘My learning process’ in the centre. They selected relevant factors, placed them on the chart, and drew red or green arrows to indicate how each factor hindered or helped their learning, respectively. The closer a factor was to the centre, the greater its impact. Blank cards were available for new factors. After creating their Pictor-chart, trainees were interviewed to explain and clarify their chart. Next, they adjusted the chart to reflect their first-year training experiences, marking rearranged sticky-notes with a dotted line and adding blue arrows for changed relationships. They were interviewed briefly about the adjusted chart.

All interviews were recorded, transcribed verbatim, and pseudonymised by an independent transcription agency.

### Participants

Potential participants received an invitation via e-mail from the main researcher (BT). To start data collection, a sample of trainees with initial (1–3 months), intermediate (1 year) and maximum (3 years) experience with EPAs was planned. As summarised in Table 1, in total 10 trainees participated. We could not include trainees with initial experience.

**Table 1:** List of interviewed trainees.


# INTERVIEW	GENDER (MALE/FEMALE)	MONTHS IN TRAINING

1	F	12 months

2	F	7 months

3	F	20 months

4	F	9 months

5	F	9 months

6	M	27 months

7	M	16 months

8	F	25 months

9	M	Recently finished

10	F	Recently finished


### Data analysis

Data were analysed in an iterative, constant comparative process. Initially detailed data units were coded (open coding) by BT. By axial coding, BT identified factors related to the learning process (Table 2). A factor was defined as a feature or action attributed to learning – as competency development – that could be stimulated or hindered through (use of) EPAs. Open codes and factors were regularly discussed in the research group. We could not define additional factors after 8 interviews. Finally, two PICTOR interviews were applied to further clarify how trainees self-regulated their learning. Each consecutive interview was compared with previous interviews for new emerging data by re-reading interviews (constant comparison) [29,30]. For interviews following the initial ones, purposive sampling was continued until theoretical sufficiency was achieved [30,35]. Theoretical sufficiency was reached when no new insights or contributions to the constructed theory occurred, according to consensus judgement of the research team (NS, LF, RS, BT). After constructing theory, representative quotes for reporting were selected by the research team [36]. For publication purposes, quotes were translated from Dutch to UK English with the aid of DeepL [37].

**Table 2:** Interactions between EPAs and factors.


EPA’S HELPING INFLUENCES	FACTOR	EPA’S HINDERING INFLUENCES

EPAs inspire to formulate learning goals and provide a framework to focus on relevant goals *Interview 3: ‘So I thought it was nice in itself or something, if you don’t really know where your learning goals are, that you start looking at what are things?’*	**Learning goal**	Conflict with other personal learning goals distracts from personal needs *Interview 4: ‘But I still had some other learning objectives. Discussing metacommunication, that was something I wanted to practise specifically. Yes, I couldn’t link that to an EPA like that much.’*

EPA descriptions provide a framework to find resources and guide study on relevant subjects *Interview 4: ‘Well, I do go through the guidelines that are recommended for the EPA, yes. So I do a bit of self-study at home, anything I don’t understand or if I think I have done or seen very little, I look it up. Yes.’*	**(Self-)study**	More work in addition to other activities gives a feeling of overload *Interview 10: Yes, and also when you see with some EPAs then hey, they say you have to do this and do that and read through that file and make this thing and then all in all you are, yes…. Yeah, that sometimes it’s too much..’*

EPAs provide a framework and content for in-depth debriefing of experiences from work. *Interview 9: ‘That it [EPA] also gave inspiration with that, let me put it this way, to further explore certain issues or have extra attention to that in a debriefing session.’*	**Debriefing session**	Feeling the need to discuss EPAs hinders spontaneity and the ability to respond to daily encountered problems. *Interview 3: ‘Especially in the first six months of training, there is so much going on that there is not much room for it [EPA]. Then you are actually mostly busy with, yes, just letting it happen to you*.’

Collecting entrustment decisions gives a feeling of progress towards becoming entrusted professional. EPAs that provide clear and relevant goals motivate to discuss and study. *Interview 6: ‘At least that my trainer has confidence in me that apparently I can do that bit well. That does feel like a confirmation or a completed part, there is apparently a requirement or a feature of a GP and I possess it. That does feel like something positive.’*	**Motivation**	Amount of work, and scholastic feel of externally imposed assignments frustrates motivation, especially in the first year where trainees experience less room to make personal choices *Interview 6: ‘Surely it was a bit the must, must character, more the scholastic character. And therefore the feeling, we have to add that too. While it already felt very much at the beginning, we already have to do a lot, …. I did think that was the negative character..’*

EPAs provide a framework to actively search for specific patients and problems. Trainees use EPAs to schedule specific patients during consultations and visits *Interview 3: ‘Yes, it is good to be aware of the different patient categories that you need to know about. Because if you just do your consultations, I discovered that very large groups are underexposed..’*	**Patients/case mix**	Impossibility to find/schedule specific patients, based on EPAs hinders planning. *Interview 4: ‘But at the same time, I also think, it just depends on what comes your way. You can think, I want to complete this EPA this year, and then have the misfortune not to come across such a case, not in practice at any point. That makes it a bit more difficult..’*

Trainees use EPAs to draw attention to specific learning goals which helps the supervisor to create opportunities and to focus and provide task-oriented feedback. *Interview 6: ‘In the first year, I just really literally had a diary that I kept online by which I kept track of topics for debriefing sessions, which she could also see. So she could then also prepare for that.’*	**Supervisor**	Supervisors that are insufficiently informed about EPAs or unwilling to use EPAs at the workplace do not respond adequately to cues from trainee, which is frustrating. *Interview 10: ‘… I also notice some resistance on the part of the supervisors, more like they don’t quite know what to do with it and what the point is.… Yes, it’s not that it’s something they, uh, it has to come from you too, of course, but it’s not that they say, oh, shouldn’t we have another go at the EPAs, there’s not an intrinsic motivation there, at least among my supervisors.’*


### Reflexivity

Researcher backgrounds influence data collection, extraction, analysis, and interpretation in qualitative research [28,30,36]. The following contextual information is considered relevant to better understand how the research team shaped the research process. BT has been involved in the development and implementation of EPAs. NS and CF have clinical and educational research backgrounds, focusing on workplace learning. RS specializes in educational research on workplace learning and qualitative research methodology. None of the team members were involved in trainee assessment, feedback, or summative decision-making. BT maintained a diary and memos to reflect on the research process and track team decisions. These were regularly reviewed by the team to ensure research findings’ integrity and the audit trail’s completeness [38].

### Ethical considerations

Aspects that have been taken into account for (ethical) approval are privacy, freedom of speech, voluntary participation, data collection, storage and access. Ethical approval has been given by the Dutch NVMO-ethical review board (NVMO-ERB 2019.6.1).

## Results

A theoretical model was developed from interviews and Pictor charts, comprising three components:

1. Factors related to Self-Regulated Learning (SRL): We identified factors attributed to workplace self-regulated learning, particularly concerning EPAs. These factors, which influence learning through EPA interactions, are summarized in Table 2, second column.
2. Helping and Hindering Influences: For each factor, we identified influences that either help or hinder learning. Helping influences are those that led to trainees gaining insights and enhancing their competency development. Conversely, hindering influences negatively impacted competency development.
3. Self-Regulation Over Time: The third component involves balancing these influences over time for effective self-regulation.

Table 2 provides a detailed description and quotes of hindering and helping influences on learning of EPAs for each factor, identified in the first stage.

Trainees experienced hindering and helping influences differently throughout the course of the training program. Trainees described how they self-regulate their learning by balancing helping and hindering influences, following three distinctive stages during placements: adaptation, taking control, and checking the boxes. Each stage appeared in both the first and final year of placements in GP-office.

During the adaptation phase, trainees focused primarily on familiarising themselves with their work and gaining control. The first year was overwhelming due to their newness at the GP office. For the final year in the GP office, returning from placements elsewhere often necessitated readjustment. There was minimal attention given to EPAs as hindering influences regarding learning goals and motivation prevailed. The use of EPAs was frequently deferred. The adaptation phase was typically shorter in the final year.

*Interview 9, Male, recently finished: ‘Yes, then I find it* [EPAs] *feels more of a hindrance at a certain point than adding anything more to me because then it feels like too much. But and then again, that also does hamper motivation on my own learning, that I think yes, where do I start? What do I still have to do all together?’*

The ‘taking control’ stage was prevalent throughout most of each placement. For most factors, helping influences prevailed. As trainees grew more comfortable, they began utilising EPAs to oversee their competence in handling key primary care issues. They identified developmental gaps linked to neglected patient groups and problems.

*Interview 6, Male: ‘Yes, kind of the concretisation of your learning plan. So that you can reflect a bit on whether or not I am on the right track. And so you can also reflect on the harder end points, where should I be after three years? I think it’s a nice way of getting a grip.’*

Trainees employed EPAs to schedule specific patients, engage in study activities, establish distinct learning objectives and discuss particular patients and issues with their supervisor. The transition from the adaptation phase could be instigated either by the trainee or through EPA discussions during the academic day. Educators and peers assisted trainees in recognising the use of EPAs and transitioning from incidental to more deliberate learning strategies. EPAs could clash with personal learning objectives, compelling trainees to balance time and effort between personal and institutional goals. In the first placement, trainees were less confident in prioritising their own learning goals, and their learning was somewhat externally dictated by the training centre’s requirements. In the final placement, trainees felt more freedom for personal choices and self-regulation, as both educators and supervisors were perceived as having less control over the trainee’s learning process. Some trainees continued to use EPAs as a framework to track competency development, while others intentionally focused on other personal learning objectives.

*Interview 9: ‘So in the first year there’s a bit more focus on it [EPAs] and you’re a bit more engaged with it and in the last year, yeah, I was mostly just trying to get as many flying hours and as many consultation encounters as possible.’*

The ‘checking the boxes’ stage typically began towards the end of each placement. In this stage, hindering influences related to learning goals, (self-)study and motivation occurred. Faced with a high-stakes progress decision, trainees recognised the necessity to acquire entrustment decisions. Although EPAs were introduced as learning tasks, with trainees having the freedom to select their own focus, obtaining statements of entrustment was often perceived as mandatory and as the most bureaucratic aspect of EPAs. Trainees understood the need for entrustment statements but found them minimally useful for self-regulation. Most trainees initiated the collection of entrustment statements by suggesting completed EPAs to their supervisor, who generally agreed with the trainees’ assessments by approving these statements.

*Interview 6: ‘And with a proposal, which I had then sent to [supervisor] with an explanation attached which I did write myself. With ‘I think the trainee is competent in this and this and this’. … And then I’d send it to [supervisor], who would look at it and adjusted it or signed it. That’s how it looked practically, actually.’*

## Discussion

Trainees self-regulate their learning at the workplace by counterbalancing helping and hindering influences of EPAs on the learning process. These influences are balanced differently across three distinct stages during primary care practice placements. The majority of EPAs’ positive influences on learning are evident in the taking control stage which constitutes the bulk of each placement. These findings suggest that EPAs, as a beneficial framework, can enhance focus and depth of learning, and support self-regulated learning at the workplace, provided trainees can effectively balance helping and hindering influences of EPAs on their learning process.

Our findings confirm expectations described by Bonnie et al., where EPAs were anticipated to offer insights into learning needs, target feedback, and identify competence development gaps [6]. Our study found EPAs useful in identifying such gaps and for planning further learning. Sagasser et al. study revealed how postgraduate GP-trainees regulate their learning via short or long self-regulation loops. Short-loop learning involved simple actions for low-complexity problems, while long-loop learning involved complex problems, with trainees setting goals and planning long-term learning activities. Feedback and external assignments helped to identify developmental gaps [14]. These long-loop characteristics align with our study’s findings, suggesting EPAs may promote long-loop learning. EPAs, when used as a framework to identify gaps and to inspire new learning goals, may enhance long-loop learning by drawing attention to blind spots relevant for competency development (intentional learning). Thus, EPAs could provide focus in a workplace learning setting where focus can be easily lost [11,12]. Our results suggest that focus may also contribute to additional depth of learning. We found that trainees used EPAs to add content and depth to debriefing sessions with their supervisors. Based on Teunissen et al [10] and Strand et al [39], discussing workplace experiences with supervisors is crucial to convert experiences into knowledge. These mechanisms may explain why trainees attributed helping influences to the use of EPAs in debriefing sessions. During debriefing sessions, trainees discuss their work and experiences with their supervisor. When EPAs are used as a framework to convert experiences into knowledge, they can contribute to deep learning from workplace experiences.

In contrast to the positive influences of EPAs on learning, trainees also reported hindrances. The attainment of entrustment decisions and the perceived obligatory use of EPAs were seen as academic, bureaucratic, and thus demotivating. These factors added to the trainees’ perceived workload of the training programme. These perceptions seem contradictory to the design in our case, where EPAs were introduced as elective learning tasks to be deployed based on trainee’s needs, whit the entrustment decision as a formative assessment to mark completion of EPAs as learning tasks. These findings suggest a gap between theory and reality related to alignment between learning objectives, learning tasks, and assessment or evaluation [40,41]. If this is the case, poor alignment of EPAs, particularly from an assessment perspective, could limit trainees’ ability to balance the stimulating and hindering influences of EPAs on learning. Consequently, a concern is that entrustment of EPAs risks becomes merely a qualification tool [42]. This could result in reduced value of EPAs to inform learning and to bridge the gap between competencies and actual work.

Our study indicates that the use of EPAs to foster self-regulated learning in a clinical setting necessitates a careful balance of their influences on self-regulated learning. The utility of EPAs evolves during placements, proving most beneficial when placements allow trainees sufficient time to transition from the ‘adaptation phase’ to the ‘taking control’ phase. Strategies should aim to minimize the duration of the ‘adaptation’ and ‘checking the boxes’ phases. In all stages, however, trainees, supervisors and teachers may experience tensions between helping and hindering influences, which are interdependently related to factors, identified in our study. Supervisors and teachers can assist trainees in managing these tensions. A recent study demonstrates how ‘Polarity Thinking’ can help stakeholders manage the balance between accountability and learner agency. Regular discussions and evaluations of interdependencies can help maintain a balance between learning and assessment goals [43]. Although studied in the context of portfolio use in programmatic assessment, we think these findings are transferable to the use of EPAs for learning and assessment.

So far, this discussion assumes that helping and hindering influences of EPAs can be balanced depending on how EPAs are implemented. But what if it is in the nature of EPAs themselves that hindrances occur and overshadow helping influences? From a sociomaterialist perspective the need to underpin statements of entrustment with multiple documented observations may very well induce a learning climate where collecting proof becomes a goal instead of a means [44,45]. In addition, EPAs are intrinsically linked to outcome-based education (OBE). OBE is increasingly challenged in the literature, as it may conflict with the higher goals and aim of medical education [7,46,47,48,49,50,51]. From this perspective, hindrances may continue to occur or even prevail over beneficial effects. Consequently, we argue to critically evaluate both the implementation of EPAs and the concept of EPAs itself.

Future research should enhance our theory on self-regulated learning by investigating additional elements such as e-portfolios, assessment tools, supervisors, and teachers. This could validate our theory through data triangulation. The roles of teachers and peers in phase transitions and balancing interactions warrant further exploration. Cognitive apprenticeship theory and the concept of scaffolding seem to resonate well with the observed differences between the first and final year [52]. Future study could explore if scaffolding is a useful approach to assist trainees in balancing interactions of EPAs with context factors.

## Limitations and Strengths

The credibility of our study could have been affected by BT’s role as interviewer and analyser. BT’s deep understanding of the GP-trainee program, often seen as beneficial for interviewing subjects, was deemed suitable by the research team [28]. Yet, there is a risk that certain patterns or habits might be overlooked or accepted as part of the program’s norms, leading to blind spots and skewed interpretation.

Theoretical sufficiency may not have been fully achieved. Experiences from trainees in early stages of their placements could have been missed, related to findings that trainees use EPAs to progress from incidental to intentional learning. As most trainees were in the programme for at least seven months, recollection bias may have caused this early phenomenon to be less remembered by trainees. Additionally, only two PICTOR interviews have contributed to our theory. PICTOR interviews were mainly added as confirmatory. Although analysed iteratively with all other data, we cannot exclude the possibility that more PICTOR interviews might have contributed to the theory. However, based on the team’s stance on theoretical sufficiency, no substantial extension of the theory was expected. A major strength of this study is that we explored actual experience with the use of EPAs for self-regulated learning, while other studies mostly described theoretical or anticipated benefits or explored aspects like development, assessment and entrustment [3,5,6,15,27,53,54,55,56,57,58,59,60]. Although limited to a single case, findings of our study can contribute to the academic field of knowledge as we provided a thick description of context and paid careful attention to reflexivity. Measures were taken to limit the impact of blind spots and skewed interpretation of data by discussing memos, codes and theory with a research team consisting of field experts from both inside and outside the training program. These measures contribute to transferability and confirmability of findings [61].

## Conclusion

EPAs can be of use to inform training and learning activities at the work place and provide a useful framework for self-regulated learning. Trainees balance helping and hindering influences of EPAs on their learning process. To profit most from the beneficial impact of EPAs on learning requires creating the right circumstances by all stakeholders involved. Results from our study indicate that it might be more favourable to entrust our trainees in using EPAs for learning and less for assessment purposes Both implementation of EPAs and the concept of EPAs itself require continuous and critical evaluation.

## Appendix 1: Interview guide

Introduction:

- Welcome participant
- Brief explanation of study and goal
- Explanation of anonymity, there are no wrong answers, stop whenever possible.
- Check for remaining questions regarding study and informed consent
- Sign informed consent

Initial question:

- What are your experiences so far with EPAs?
- Transient question.
- Explore experiences so far in depth, how started, made any agreements e.g. with supervisor.

Core questions:

- How did you use EPAs when formulating learning goals?
- How did you use EPAs to collect feedback?
- What does an entrustment decision mean for you?
- What EPAs do you consider useful? Why?
- What EPAs do you consider useless? Why?

Ending questions:

- To wrap this up, what are, based on you experiences disadvantages of EPAs?
- End what do you consider advantages of EPAs?
